# Salivary Testosterone and Sexual Function and Behavior in Men and Women: Findings from the Third British National Survey of Sexual Attitudes and Lifestyles (Natsal-3)

**DOI:** 10.1080/00224499.2021.1968327

**Published:** 2021-10-11

**Authors:** W.G. Macdowall, S. Clifton, M.J. Palmer, C. Tanton, A.J. Copas, D.M. Lee, K.R. Mitchell, C.H. Mercer, P. Sonnenberg, A.M. Johnson, K. Wellings

**Affiliations:** a Department of Public Health, Environments and Society, Faculty of Public Health and Policy, London School of Hygiene & Tropical Medicine; b Institute for Global Health, University College London, Mortimer Market Centre; c NatCen Social Research; d Department of Global Health and Development, Faculty of Public Health and Policy, London School of Hygiene & Tropical Medicine; e Faculty of Health, Psychology and Social Care, Manchester Metropolitan University; f MRC/CSO Social and Public Health Sciences Unit, University of Glasgow

## Abstract

Using data from the third British National Survey of Sexual Attitudes and Lifestyles (Natsal-3) we examined associations between salivary testosterone (Sal-T) and sexual function and behavior. Single morning saliva samples were self-collected from a subsample of participants aged 18−74 years and analyzed using mass spectrometry. 1,599 men and 2,123 women were included in the analysis (40.6% of those invited to provide a sample). We adjusted for confounders in a stepwise manner: in model 1 we adjusted for age only; model 2 for age, season and relationship status, and model 3 we added BMI and self-reported health. In the fully adjusted models, among men, Sal-T was positively associated with both partnered sex (vaginal sex and concurrent partners) and masturbation. Among women, Sal-T was positively associated with masturbation, the only association with partnered sex was with ever experience of same-sex sex. We found no clear association between Sal-T and sexual function. Our study contributes toward addressing the sparsity of data outside the laboratory on the differences between men and women in the relationship between T and sexual function and behavior. To our knowledge, this is the first population study, among men and women, using a mass spectrometry Sal-T assay to do so.

## Introduction

The role of testosterone (T) in human sexual function, desire, and behavior is an area of intense interest and investigation.

Among men, overt T deficiency − caused by pituitary or testicular disease (male hypogonadism) − is known to result in a wide range of symptoms, including erectile dysfunction and reduced sexual desire, which can be treated with testosterone replacement therapy (TRT) (Bhasin et al., 2018; Rastrelli et al., 2018, 2016). Less clear, however, is the relationship between levels of T across the normative range and aspects of sexual function and behavior. In community studies among men, T has been associated with frequency of morning erections (O’Connor et al., 2011; Wu et al., 2010), sexual thoughts (O’Connor et al., 2011; Wu et al., 2010) and masturbation (O’Connor et al., 2011). Associations with erectile function have been found in some studies (Cunningham et al., 2015; Gades et al., 2008; O’Connor et al., 2011) but not others (Marberger et al., 2011). T has also been implicated in partnering and parenting; partnered men tend to have lower levels of T compared to those who are single (Grebe et al., 2019) − a finding that is supported in longitudinal studies that have assessed T levels before and after divorce and remarriage (Holmboe et al., 2017) − and men who are fathers tend to have lower T than those who are not (Grebe et al., 2019). These findings have often been interpreted from the evolutionary perspective of the Challenge Hypothesis in which it is argued that there are trade-offs between high T and challenge, and low T and parenting (Wingfield et al., 1990). The Challenge Hypothesis infers that men with higher T will be more motivated to seek out sexual partners, may change sexual partners more frequently and have greater interest in extradyadic sex. However, the direction of association is unclear and it has also been suggested that it is not relationship status per se that is important but rather orientation toward investment in establishing and maintaining monogamous partnerships, with some evidence suggesting that men in long-term relationships who have a positive orientation to extra-dyadic sex have levels of T that are similar to men who are single (Edelstein et al., 2011).

The role of T in women’s sexuality is even less well understood. Previous research on the relationship between hormonal status and sexual behavior in women has tended to focus on aspects of female reproductive biology such as menstruation, pregnancy and menopause and often excluded T (van Anders, 2013). The ‘presumed tie’ between T and masculinity, and the predominant framing of T as ‘a driver of male reproductive tactics’ has likely influenced the focus of research (van Anders, 2013). T, however, has received more attention in recent years driven in part by the search for therapeutic solutions to problems of female sexual response. T is implicated in women’s sexuality, though few large community studies have been conducted (Davis et al., 2005; Randolph et al., 2015). The clinical significance of ‘low T’ and the role of TRT in treating low sexual desire, however, is subject to ongoing debate with some suggestion that the focus on T is misplaced and it should rather be on estrogen (Cappelletti & Wallen,2016).

It is well established that sexual function and behavior are influenced by social factors (Baumeister et al., 2001) and the strength of this influence appears to be greater among women than men (Bancroft, 2009; Baumeister et al., 2001). Important gender differences in the role of T in sexual desire and response have also been posited (Bancroft & Graham, 2011). It has further been suggested that the moderating effect of social factors on the influence of hormonal status on sexual function and behavior may be greater among women than men (Pringle et al., 2017; van Anders, 2012), though this has rarely been examined outside of the laboratory.

The challenges to empirical investigation in this area, and to the interpretation of findings, are many. Firstly, measures of T, and assays employed, differ between studies. In clinical research and practice, T is most commonly assessed through the collection of blood samples from which Total-T can be measured and Free-T calculated (Vermeulen et al., 1999). Free-T can be measured directly by equilibrium dialysis but this is not routinely used. Total-T includes the element that is bound to carrier proteins − specifically Sex Hormone Binding Globulin (SHBG) and albumin − plus the small proportion (~1−2%) that is ‘free’ (unbound). The bioavailability of T is influenced by levels of SHBG, which in turn varies by several factors including age, Body Mass Index (BMI), and use of hormonal contraception (Camacho et al., 2013; Wu et al., 2008; Zimmerman et al., 2014). Free-T is considered to be the biologically active fraction and hence to potentially be a better indicator of T status. In population research, salivary T (Sal-T) is an attractive alternative to serum-T, given the relative ease of sample collection. Sal-T, though not identical to serum Free-T, correlates fairly well with serum Free-T (Fiers et al., 2014; Keevil et al., 2014) and is unaffected by levels of SHBG (Keevil et al., 2016).

Secondly, there are methodological differences between studies, many of which have involved clinical or convenience samples. Where large community-based studies have been carried out, they have tended to be among older men, and have been conducted in the context of examining the impact of aging on disease processes (Cumming et al., 2009; Gray et al., 1991; Lee et al., 2009). The little research that has been conducted using community samples of women (Davis et al., 2005) has faced measurement problems due to the low concentration of T in women, coupled with poor specificity of immunoassay methods (Davis et al., 2019). Important too is confounding, most notably by age and health, both of which are associated with levels of T (Davison et al., 2005; Keevil et al., 2017; Wu et al., 2008) − and its main carrier protein SHBG (Maggio et al., 2008; Wu et al., 2008) − and with sexual function and activity (Field et al., 2013; Mitchell et al., 2013).

A third challenge is presented by variation in how sexual function, desire, and behavior are conceptualized and measured in studies, and a lack of attention to psychosocial factors influencing human sexuality. Sexual behavior is a complex phenomenon that is socially constructed and operates within wider cultural structures that may limit its expression and set gendered expectations on what is ‘appropriate’ and ‘socially accepted.’ Even outwardly seemingly biological processes, such as erectile response, are known to be influenced by a complex range of psychosocial factors (Feldman et al., 1994; Rosen, 2001; Seidman & Roose, 2001), posing challenges to isolating the contribution of T.

In this paper, we analyze data from the third National Survey of Sexual Attitudes and Lifestyles (Natsal-3) to examine associations between Sal-T and aspects of sexual function and behavior. The research questions guiding the analysis focus, firstly, on whether the strength of association might vary according to the facet of sexual function and behavior being assessed, the hypothesis being that such variation might reflect the relative strength of hormonal and social influences on each. For example, in terms of sexual behavior, we hazarded that solitary sex may be more strongly associated than dyadic sex with Sal-T, given the stronger influence of social context on the latter. Secondly, we were interested in whether the strength of associations with Sal-T varied between men and women, the hypothesis being that − since social context is more strongly implicated in women’s sexual behavior − dyadic sex might be more weakly associated with Sal-T than with solitary sex among women.

## Method

### Participants and Procedures

Full details of the Natsal-3 methods, including details of the saliva sample collection and testing, are described elsewhere (Erens et al., 2013, 2014). In summary, Natsal-3 is a probability sample survey of 15,162 people (6,293 men and 8,869 women) aged 16−74 years resident in Britain. Interviews took place between September 2010 and August 2012 using a combination of computer-assisted personal interviewing (CAPI) and computer-assisted self-interview (CASI) for the more sensitive questions. The response rate was 57.7%.

Single morning saliva samples were self-collected from a subsample of men and women aged 18−74 years, who did not regularly work night shifts. Consenting participants were given a self-collection pack and asked to provide their sample before 10 am, to minimize diurnal variation in T (Keevil et al., 2014). Premenopausal women were not asked to provide their samples at any particular point in their menstrual cycle on the basis that variation in T across the cycle is relatively small compared to other sources of variation, and was not a focus of our research (van Anders et al., 2014). Participants were asked not to brush their teeth, eat or chew before giving the sample, and to spit directly into a plain polystyrene tube. Samples were posted to the laboratory where they were prepared and frozen at −80°C until analysis using liquid chromatography tandem mass spectrometry (LC-MS/MS). The LC-MS/MS Sal-T assay was developed using strict validation criteria (Keevil et al., 2014), with a lower limit of quantification of 6.5 pmol/L. Full details of the laboratory methods, including the validation of the assay, have been published elsewhere (Erens et al., 2013; Keevil et al., 2014).

Altogether, 9,170 eligible participants were invited to provide a saliva sample, 6,515 (71.0%) agreed to do so and 4,591 samples were received by the laboratory and matched to the survey data (50.1% of those invited). Four hundred and sixty-three samples were excluded due to issues with sample quality (Keevil et al., 2017) leaving 4,128 participants (45.0% of those invited) with a testosterone result (1,675 men; 2,453 women). Overall, there was no difference in the proportion of men and women with a useable T result (data not shown); the higher number of women included in the analysis reflects the higher number of women in the Natsal sample as a whole. Participants who reported clinical conditions or taking medication likely to affect testosterone levels were excluded from the analysis (currently taking medication for epilepsy (15 men; 15 women) or prostate disease (43 men); treatment in the past year for an ovarian, testicular, or pituitary condition (16 men; 23 women) or for polycystic ovaries (35 women); pregnant at interview (42 women); current receipt of hormone replacement therapy (HRT) (62 women); ever receipt of HRT together with having had a hysterectomy (proxy measure for having had ovaries removed; 181 women); missing data for these questions (3 men; 15 women)) resulting in 1,599 men and 2,123 women being included in the analysis. These exclusions aimed to minimize confounding of the relationship between testosterone and sexual function and behavior caused by these factors which are known to influence testosterone levels. Women taking hormonal contraception (oral contraceptive pill, Mirena coil, injections, implants, or the contraceptive patch) in the past year were included in analyses to avoid biases possibly resulting from excluding this substantial proportion of women (29% of all women with a valid saliva sample, but up to 73% of women in the youngest age group (18−24 years)). However, additional sensitivity analyses were carried out excluding these women, to assess the extent to which their inclusion affected associations with sexual function and behavior.

### Measures

Variables selected for this analysis included capacity for sexual expression, that is, aspects of sexual function. We also included measures of solitary expression, that is, masturbation and of partnered sexual expression and sexual attitudes.

### Sexual Function Measures

Sexual function was assessed using the Natsal-SF, a psychometrically validated 17 item (16 items per gender) measure comprising three components. The first component includes problems with sexual response, the second, captures sexual function in the relationship context and the third, self-appraisal of sex life. Participants who had at least one sexual partner in the year prior to interview were given a score on the Natsal-SF, and those in the lowest quintile of the sex-specific distribution were considered to have ‘low’ sexual function (see Mitchell et al., 2012 and Jones et al., 2015 for details of the measure and its scoring). We also used a number of individual items from within the Natsal-SF. Using the past year as the reference period, participants who had at least one sexual partner in that time were asked if they had experienced any of the following for a period of three months or more: lacked interest in having sex; lacked enjoyment in sex; had an uncomfortably dry vagina (women only) and had trouble getting or keeping an erection (men only). In the self-appraisal component of the measure, participants who had *ever* been sexually active, were asked to respond to the statement *“I feel distressed or worried about my sex life”* we considered those who agreed, or agreed strongly, with this statement as being distressed. ‘Sex’ was defined as vaginal, oral, or anal intercourse with an oppositesex or same-sex partner, and ‘sex life’ as sexual thoughts, sexual feelings, sexual activity, and sexual relationships.

### Sexual Behavior and Attitudinal Measures

We looked at a range of sexual behavior measures over three different time periods. We measured frequency of sex and engaging in different sexual practices, namely, vaginal sex, receiving oral sex, giving oral sex, anal sex, and genital contact without intercourse in the *four weeks prior to interview*.We measured number of sexual partners; concurrent (overlapping) partners; reporting a same sex partner and paying for sex (men only*) in the past five years*. Number of partners and ever having same sex experience (with genital contact) were measured *over the lifetime*. Other measures included in the analysis were: recency and frequency of masturbation; sexual attraction (opposite sex only, or any same sex) and attitudes toward different sexual behaviors. The attitudinal questions were asked in the CAPI section of the questionnaire, after the CASI, with the use of showcards. First, participants were asked their views about different types of sexual relationships including *“A married person having sexual relations with someone other than his or her partner?*” and “*A person having one-night stands?*” (response options were: Always wrong; mostly wrong; sometimes wrong; rarely wrong; not wrong at all and depends/don’t know). Next, participants were asked how far they agreed, or disagreed, with a number of statements including: “*It is natural for people to want sex less as they get older*” and “*Men have a naturally higher sex drive than women*” (response options were: Agree strongly; agree; neither agree nor disagree; disagree; disagree strongly and don’t know). The full Natsal questionnaire is available at http://www.natsal.ac.uk/natsal-3/questionnaire.aspx.

### Statistical Analyses

Statistical analyses were carried out using STATA (version 13.1) accounting for the complex survey design (stratification, clustering, and weighting of the sample). We applied weighting to correct for unequal probability of selection and differential response (by age, sex, and region) to the survey itself; and to correct for unequal probability of selection and differential response to the saliva sample. The factors we found to be associated with providing a saliva sample included age at interview, ethnicity, self-reported general health, and sexual function; the saliva weighting significantly reduced these biases (Erens et al., 2013).

Throughout, we censored very high Sal-T values so that, for each 10-year age group stratified by sex, values above the 99th percentile were assigned a value equal to that of the 99th percentile. The Sal-T data for men were normally distributed; however, the distribution for women was positively skewed and so values were transformed on the natural log scale for analysis. Accordingly, for men we present linear regression coefficients representing differences in mean testosterone in pmol/l, whereas for women we present ratios of geometric mean Sal-T obtained from exponentiated coefficients. Interval regression was used to assign values to the range 0 to 6.5 pmol/l for 3 men, and 0.5 (to allow log transformation) to 6.5 pmol/l for 62 women with testosterone levels below the limit of detection (<6.5 pmol/l) (Clifton et al., 2016; Keevil et al., 2017).

Descriptive statistics are presented as mean T (standard error), with multivariable linear regression used to assess differences in mean T by the sexual function or behavior variables of interest.

In our earlier analyses, we identified a number of factors that were significantly associated with mean Sal-T levels that may confound the relationship between Sal-T and sexual function and behavior (Clifton et al., 2016; Keevil et al., 2017). In summary, among both men and women mean Sal-T decreased with increasing age, and seasonal variation was observed (with mean Sal-T lowest in the summer for men and highest in the summer for women). Among men only, we found variation in mean Sal-T by relationship status independent of age, with the highest levels among those who were not currently in a steady relationship, and lowest levels among those who were married or cohabiting. Also among men only, and independent of age, we found negative associations between mean Sal-T and BMI and self-reported general health. In the current analysis, to assess how these potential confounders affected the associations − and to determine whether any aspects of sexual function and/or behavior were associated with Sal-T independent of these factors − we ran a number of multivariable linear regression models. In the first model, we adjusted only for age, using both linear and quadratic terms to account for a non-linear relationship of testosterone with age (Keevil et al., 2017). In the second, we adjusted for age and additionally for season and relationship status. Lastly, we added the key health factors previously identified (Clifton et al., 2016) − BMI and self-reported general health − to the models. In this way, any identified associations between Sal-T and sexual function and behavior would not be explained by these confounding factors.

### Ethics

The Natsal-3 study was approved by the Oxfordshire Research Ethics Committee A (reference: 10/H0604/27). Written informed consent was obtained for anonymized testing of saliva samples, without return of results.

### Findings

Mean Sal-T was higher among men than women (223.5 pmol/L and 37.1 pmol/L respectively) and differences in associations with Sal-T and sexual behavior were observed between the two (Tables 1 and Tables 2).

### Sexual Function

In the unadjusted analysis, Sal-T was lower in men who reported erectile difficulties and women who reported experiencing an uncomfortably dry vagina (for at least three months in the past year) but after adjustment for age (model 1) these associations did not persist. In both instances, the additional adjustments in models 2 and 3 made little difference to the associations, pointing to age as the key confounder.

No association was observed, in either men or women, between Sal-T and overall low sexual function measured using the Natsal-SF or between Sal-T and the individual problems of sexual response we investigated (i.e., lacking enjoyment in sex, distress about sex life, and, among men, lacking interest in sex). Among women, there was a significant association between Sal-T and reporting lacking interest in sex in the age-adjusted model (model 1) but this was attenuated after further adjustments for relationship status, season, BMI and general health status (model 3). In the fully adjusted model the geometric mean ratio was 0.92 (95% confidence interval 0.84, 1.00; *p* =.0592) for women reporting lacking interest in sex (for at least 3 months in the past year) compared to those who did not.

### Sexual Behavior and Attitudes

Among men, in terms of partnered sexual behavior, the strongest association with Sal-T was with reporting concurrent − that is, overlapping − sexual partners. The linear regression coefficient in the fully adjusted model (model 3) for those reporting concurrent partnerships in the past 5 years compared to those who did not was 20.87 (4.47, 37.26; *p* =.0127). This was followed in strength of association by vaginal sex and receiving oral sex from a partner (adjusted coefficients 13.44 (1.53, 25.35; *p* =.0271) and 11.20 (−0.05, 22.46; *p* =.0510)), respectively, for those reporting these sexual practices in the past four weeks versus those who did not. Higher levels of Sal-T were also associated with recency and frequency of masturbation. Men who had masturbated longer than a year ago had lower mean Sal-T compared to men who had masturbated more recently; adjusted coefficient −21.82 (−36.97, −6.67; *p* =.0269) (for last occasion of masturbation longer than a year ago, compared to the last 7 days).

Among men, a weak association was also observed between higher Sal-T and having had a same-sex partner in the past 5 years (adjusted coefficient for same-sex partner in the past 5 years versus not: 22.30 (−0.75, 45.34; *p* =.058)), though the proportion reporting a same-sex partner in the past 5 years was low (3.0%, (2.2%, 4.0%)). Significant associations were also seen between Sal-T and two attitudinal statements: acceptance of one-night stands and of non-exclusivity in marriage, with men endorsing these more permissive attitudes to sex having higher mean Sal-T than those who did not.

Among women, Sal-T was most strongly associated with masturbation and the association was stronger than seen among men. Women who had masturbated longer than a month ago had lower mean Sal-T compared to women who had masturbated more recently; adjusted geometric mean ratio 0.84 (0.75, 0.95; *p* =.0077) (for last occasion of masturbation longer than 4 weeks but less than a year, compared to the last 7 days). Frequency, as well as recency, of masturbation was associated with Sal-T in women; mean Sal-T was higher in women who had masturbated on two or more occasions in the last 7 days compared to those who had masturbated only once (adjusted geometric mean 1.24 (1.05, 1.46; *p* =.0009)). Sal-T was also significantly higher among women reporting ever experience of same-sex sex compared to those who did not (1.15 (1.01, 1.31; *p* =.0378)). In the sensitivity analysis, in which we excluded women who had used hormonal contraception in the last year, these associations were attenuated but remained significant.

## Discussion

To our knowledge, this is the first population level study, of both men and women, using a validated salivary measure to explore the associations between Sal-T and aspects of sexual function and behavior.

We found no clear associations in our data between Sal-T and either overall sexual function (as measured by the Natsal-SF) or individual problems with sexual response in men or women. Among women, our data showed solitary sex to be more strongly associated than partnered sex with Sal-T; levels of Sal-T were higher in those who masturbated more recently and more frequently. We found no association between Sal-T and heterosexual partnered sexual activity among women, as measured by occurrence of vaginal sex in the past month, and nor did we find an association with number of partners or concurrency. The only measure of partnered sex associated with Sal-T among women was ever experience of same-sex behavior.

Among men, Sal-T was associated with masturbation but not *more* strongly than it was with partnered sex. Associations were seen between higher levels of Sal-T and recent occurrence of heterosexual partnered sex and with concurrency of sexual partners in the last five years, but not with number of sexual partners. The association with concurrency was reflected in men’s attitudes toward ‘casual’ sexual encounters, which were similarly linked with higher levels of Sal-T.

### Contextualization and Interpretation

The absence of an association between T and overall sexual function in men in our large dataset is unsurprising given the measure of overall sexual function used in Natsal-3 which, as indicated above, took account not only of individual problems with response, but also the relational context, which is heavily influenced by psychosocial factors. The absence of any association with individual aspects of sexual function (erectile difficulties, lacking enjoyment in sex, distress about sex life, lacking interest in sex) is perhaps more surprising. The dominant narrative assumes T is the ‘biological driver’ of sexual desire in men. The fact that men have both higher levels of T and report higher levels of interest in sex than women seems to speak to this narrative (van Anders, 2012). Much of the evidence linking T with sexual desire in men has, however, come from clinical studies among those with overt T deficiency in the context of investigating the effects of TRT (Corona et al., 2017). There is little empirical evidence (van Anders, 2012), including that now provided by our study that T levels in men within the normal range are associated with sexual desire. In the European Male Aging Study (EMAS), which focused specifically on older men − though like Natsal drew on a large sample of community dwelling individuals − only weak associations were found between aspects of sexual function and T. These included ‘overall sexual function’ (O’Connor et al., 2011) and erectile dysfunction and frequency of both sexual thoughts and morning erections, though the associations with these latter three sexual symptoms were attenuated when adjustments were made for age, BMI, and co-existing health conditions (Wu et al., 2010). Further, the findings from EMAS highlight the non-linear relationship between T and aspects of sexual function and point to symptom-specific T ‘thresholds’; only under the ‘threshold’ does the probability of experiencing the sexual symptom increase (O’Connor et al., 2011; Wu et al., 2010). Hence, among older men, androgen deficiency is only likely to be a key pathogenic component in problems of sexual function when T levels are overtly subnormal (Wu et al., 2010). In older men with unequivocal age-related hypogonadism, TRT has been associated with modest improvements in sexual function (Matsumoto, 2019; Snyder et al., 2016). Evidence of the value of T supplementation for ‘low T’ within the normal range as a therapeutic solution to problems, such as erectile dysfunction and low libido, however, is lacking (Huo et al., 2016).

The few large community studies that have been conducted in women have identified associations between androgens and sexual function though in unadjusted analyses (Davis et al., 2005), or among women in menopausal transition (Randolph et al., 2015). In our unadjusted model, we did find an association between Sal-T and sexual desire in women, which remained significant after adjustment for age (with women lacking interest in sex having lower Sal-T than those who did not) but was attenuated after further adjustments for relationship status, season, BMI, and general health status, highlighting the importance of contextual factors. The current global consensus is that there is insufficient evidence regarding the use of T for the treatment of sexual function in premenopausal women, but among postmenopausal women T may yield benefits in terms of increasing sexual desire (as well as other components of sexual function including arousal and orgasmic function) (Davis et al., 2019). Evidence from controlled trials among postmenopausal women indicates that estrogen-only therapies are also associated with increases in sexual desire and that these effects can be enhanced when estrogen is coupled with T (Cappelletti & Wallen, 2016).

Our data support our prior assumption that the relative influence of hormonal status and social context, and hence the strength of associations between Sal-T and sexual behavior, would vary between men and women. Attempts to understand why dyadic sex, especially partner concurrency, is more strongly associated with T among men than women have drawn on evolutionary theories asserting that it may have greater reproductive advantage for men (Puts et al., 2015; van Anders et al., 2015). Yet associations between T and dyadic and solo sex may also be differentially moderated in men and women by gendered social norms regulating sexual behavior (van Anders et al., 2015). Variation in the extent to which men and women may be differentially socialized to non-exclusivity features regularly in explanations as to why men report larger numbers of sexual partners than women in research (Jonason & Fisher, 2009; Mitchell et al., 2019).

Sal-T’s marked link with masturbation among women, in the absence of an observed link with aspects of partnered behavior, may be seen as consistent with the notion of a stronger moderating effect of social factors on hormonal influences on women’s behavior. It has been proposed that masturbation may be a ‘truer’ measure of sexual desire, as although socially censured, it is neither constrained by social surveillance nor dependent on social relations. The suggestion in our data of a stronger link with solitary than partnered sexual activity among women accords with evidence reported elsewhere; albeit from either laboratory studies and/or those utilizing smaller convenience samples (Randolph et al., 2015; van Anders, 2012). Interpretation of these findings has drawn on the bi-directionality of the association between T and sexuality (Goldey & van Anders,2011) and on the different meanings and motivations attached to solitary and partnered sex. For example, qualitative research among women points to solitary sexuality as primarily erotic and partnered sexuality as nurturant (Goldey et al., 2016). Women self-identifying as heterosexual have been shown to be more likely to reach orgasm in solitary compared with partnered sex (Carvalheira & Leal, 2013) and the experience of orgasm has been found to increase levels of T (van Anders et al., 2007).

Our finding of higher mean Sal-T in women with ever experience of same sex sex is illuminated by a recent systematic review, investigating whether lesbian and bisexual women may have different levels of sex hormones compared to heterosexual women. The review found tentative evidence of higher T among sexual minority women, though the heterogeneity of studies and problems with confounding made it hard to draw definitive conclusions (Harris et al., 2020).

### Strengths and Weaknesses

This study had a number of strengths. Firstly, Natsal-3 is a large population-based study of men and women, covering a wide age range and capturing multiple aspects of sexual function, behavior, and attitudes. Secondly, Sal-T was measured by the ‘gold standard’ method of mass spectrometry using samples collected at the same time of day in order to account for the diurnal variation in testosterone. Thirdly, we were able to adjust for known confounders identified in our earlier analysis (Clifton et al., 2016; Keevil et al., 2017), so that independent associations between Sal-T and sexual function and behavior could be established. A number of limitations need also to be considered. Firstly, nonparticipation bias is likely to have occurred both in relation to recruitment to the main survey and providing a saliva sample. There were known differences between those who did and did not return a saliva sample, though statistical weighting was used to minimize these biases. The second limitation is that, with the exception of items relating to appraisal of sex life, the Natsal-SF (which included the questions about the individual problems with sexual response) was only asked of people who were sexually active in the past year and so excluded those who may not have had sex in over a year because of sexual difficulties. The third limitation relates to the adjustments made. While we did adjust for variables identified from our previous analyses as linked with both Sal-T and sexual function and behavior (Clifton et al., 2016; Keevil et al., 2017) there are, however, likely to be other confounders that we have not adjusted for. A further limitation relates to the complexity of the phenomena under investigation and the challenge in establishing causal direction when using cross-sectional data and single saliva samples given evidence that the relationship between T and sexual behavior is bi-directional (Escasa et al., 2011). We also have to recognize the limitations of a peripheral measure of T in assessing T status. In men and women, it is thought that a large proportion of androgens (and estrogens) are produced *within* cells where they exert their action and circulating androgens do not reflect this ‘intracrine’ androgen synthesis (Labrie, 1991). Relatedly, different forms of the androgen receptor are thought to vary in their sensitivity to T (Wåhlin-Jacobsen et al., 2018). Hence, circulating T is only part of a complex picture.

Our study contributes toward addressing the deficit in terms of attention paid to the role of T in women’s sexuality (Bancroft & Graham,2011) and the sparsity of data on the differences between men and women in the relationship between T and sexual function and behavior. Our data tend to confirm that differences between men and women need to be understood by examining them in the context of both social and hormonal influences on sexual function and behavior.

## Figures and Tables

**Table 1:** Associations between mean Sal-T and sexual behaviors and sexual function among men.

	% of sample [weighted]												*Denominators*	
	%	95% CI	Mean Sal-T Pmol/L	*SE*	Crude Coeff.*	95% CI	adjusted Coeff. 1*	95% CI	adjusted Coeff. 2*	95% C.I	adjusted Coeff. 3*	95% C.I	*unwt*	*wt*
All men	100%		223.5	*3.33*	-	-	-	-	-	-	-	-	*1599*	*1866*
**Sexual function**														
Problems achieving/maintaining an erection for at least 3 months in past yr^														
No	86.8	[84.6, 88.7]	233.5	*3.90*	-	-	-	-	-	-	-	-	*1010*	*1315*
Yes	13.2	[11.3, 15.4]	203.4	*8.39*	−28.60	[−46.53,−10.67]	1.21	[−15.65,18.08]	0.55	[−16.34, 17.44]	1.73	[−15.44, 18.89]	*198*	*200*
						p =.0018		p =.888		p =.949		p =.843		
Lacked interest in having sex for at least 3 months in past yr^														
No	84.8	[82.2, 87.0]	229.7	*3.90*	-	-	-	-	-	-	-	-	*1011*	*1285*
Yes	15.2	[13.0, 17.8]	228.5	*8.89*	−2.77	[−20.01, 14.48]	0.98	[−13.78,15.74]	2.53	[−12.05, 17.12]	3.55	[−10.29, 17.38]	*197*	*231*
						p =.753		p =.897		p =.733		p =.615		
Lacked enjoyment when having sex for at least 3 months in past yr^														
No	95.2	[93.3, 96.6]	229.2	*3.70*	-	-	-	-	-	-	-	-	*1156*	*1442*
Yes	4.8	[3.4, 6.7]	235.7	*15.00*	9.48	[−21.40,40.36]	−2.10	[−30.62, 26.41]	0.01	[−28.13, 28.14]	−2.50	[−29.28, 24.28]	*52*	*73*
Distressed or worried about sex life: agree strongly/agree						p =.821		p =.876		p =.747		p =.864		
No	88.6	[86.6, 90.3]	224.9	*3.49*	-	-	-	-	-	-	-	-	*1354*	*1602*
Yes	11.4	[9.7, 13.4]	227.1	*9.85*	−2.28	[−22.02, 17.46]	−1.38	[−18.73,15.96]	−2.87	[−20.25, 14.52]	1.46	[−15.27, 18.20]	*196*	*206*
						p =.547		p =.885		p =.100		p =.855		
Overall sexual function^														
Normal	79.8	[77.0, 82.2]	232.1	*4.06*	-	-	-	-	-	-	-	-	*929*	*1214*
Low	20.2	[17.8, 23.0]	219.5	*7.71*	−10.57	[−27.36, 6.23]	−0.47	[−15.52,14.58]	−0.54	[−15.38, 14.30]	0.72	[−13.67, 15.12]	*283*	*308*
						p =.218		p =.951		p =.943		p =.921		
**Sexual behavior**														
*Masturbation*														
Last occasion of masturbation														
In last 7 days	49.7	[46.4, 52.9]	244.3	*4.36*	-	-	-	-	-	-	-	-	*746*	*917*
Between 7 days and 4 weeks	17.9	[15.7, 20.2]	216.5	*6.38*	−29.11	[−44.20,−14.02]	−6.05	[−20.40, 8.29]	−4.38	[−18.88, 10.12]	−6.26	[−20.43, 7.92]	*290*	*244*
Between 4 weeks and 1 year	15.2	[12.9, 17.9]	218.1	*10.08*	−33.52	[−50.65,−16.39]	−1.15	[−16.21,13.92]	0.16	[−15.10, 15.42]	−0.26	[−15.46, 14.95]	*239*	*215*
Longer than 1 year ago/never	17.2	[15.0, 19.7]	180.1	*7.49*	−62.88	[−79.07, −46.68]	−25.16	[−42.06, −8.26]	−23.34	[−39.89, −6.78]	−21.82	[−36.97, −6.67]	*280*	*436*
						p <.0001		p =.0248		p =.0343		p =.0269		
No. of occasions of masturbation in past 7 days														
0	50.6	[47.4, 53.9]	204.5	*4.75*	−23.22	[−41.98, −4.46]	−8.25	[−24.94, 8.43]	−7.36	[−23.90, 9.17]	−7.16	[−22.94, 8.63]	*809*	*917*
1	13.5	[11.4, 15.9]	223.9	*8.74*	-	-	-	-	-	-	-	-	*189*	*244*
2	11.9	[10.1, 13.9]	227.5	*7.06*	1.94	[−20.47, 24.34]	−7.93	[−29.04,13.17]	−8.01	[−28.87, 12.84]	−9.79	[−29.65, 10.07]	*189*	*215*
3+	24.1	[21.5, 26.8]	262.6	*6.37*	36.23	[15.17, 57.29]	9.21	[−9.21, 27.64]	7.86	[−10.52, 26.25]	9.94	[−7.38, 27.27]	*359*	*436*
						p <.0001		p =.0800		p =.1508		p =.0485		
*Sexual behavior in the past 4 weeks*														
No. of occasions of sex#														
0−2	54.9	[51.7, 57.9]	211.9	*4.16*	-	-	-	-	-	-	-	-	*903*	*964*
3−4	18.5	[16.1, 21.2]	237.0	*7.32*	25.51	[9.22, 41.79]	11.71	[−2.94, 26.35]	14.96	[−0.17, 30.09]	10.80	[−3.92, 25.51]	*234*	*325*
5+	26.6	[23.8, 29.6]	242.9	*7.33*	26.45	[11.36, 41.55]	7.00	[−6.96, 20.97]	10.12	[−4.23, 24.47]	7.59	[−6.16, 21.35]	*352*	*467*
						p =.0003		p =.2626		p =.122		p =.299		
Vaginal sex														
No	36.2	[33.5, 39.0]	215.7	*4.95*	-	-	-	-	-	-	-	-	*692*	*669*
Yes	63.8	[61.0, 66.5]	230.3	*4.43*	16.38	[3.92, 28.85]	6.33	[−4.37, 17.03]	16.10	[4.03, 28.16]	13.44	[1.53, 25.35]	*884*	*1179*
						p =.0101		p =.246		p =.0090		p =.0271		
Received oral sex#														
No	60.3	[57.3, 63.3]	212.7	*3.82*	-	-	-	-	-	-	-	-	*1024*	*1112*
Yes	39.7	[36.7,42.7]	243.8	*5.68*	27.79	[15.17,40.41]	8.69	[−2.96, 20.33]	10.29	[−1.46, 22.04]	11.20	[−0.05, 22.46]	*549*	*732*
						p <.0001		p =.1436		p =.0860		p =.0510		
Gave oral sex#														
No	59.8	[56.8, 62.8]	215.3	*3.75*	-	-	-	-	-	-	-	-	*1019*	*1103*
Yes	40.2	[37.2, 43.2]	239.4	*5.70*	22.09	[9.54, 34.64]	3.95	[−7.09, 15.00]	6.16	[−5.18, 17.50]	7.84	[−3.01, 18.68]	*555*	*741*
						p =.0006		p =.483		p =.287		p =.1565		
Anal sex#														
No	95.3	[93.8, 96.4]	223.4	*3.43*	-	-	-	-	-	-	-	-	*1504*	*1756*
Yes	4.7	[3.6, 6.2]	258.2	*12.88*	33.90	[8.00, 59.81]	15.31	[−5.93, 36.54]	14.15	[−7.21, 35.52]	15.02	[−5.37, 35.41]	*70*	*87*
						p =.0104		p =.1576		p =.194		p =.1487		
Genital contact without intercourse#														
No	53.5	[50.5, 56.6]	216.2	*4.30*	-	-	-	-	-	-	-	-	*930*	*986*
Yes	46.5	[43.4, 49.5]	235.1	*4.83*	17.97	[6.03, 29.90]	2.53	[−8.56, 13.62]	6.39	[−5.04, 17.82]	6.54	[−4.42, 17.50]	*642*	*856*
*Sexual behavior in the past 5 years*														
						p =.0032		p =.655		p =.273		p =.242		
Number of sexual partners#														
0	9.3	[7.8, 11.0]	207.7	*10.35*	-	-	-	-	-	-	-	-	*209*	*169*
1	58.0	[55.1,60.9]	210.6	*3.80*	5.78	[−15.97, 27.52]	−0.55	[−18.22,17.13]	9.28	[−10.50, 29.07]	3.49	[−16.93, 23.91]	*828*	*1057*
2	10.6	[8.9, 12.6]	244.6	*9.76*	40.95	[12.84, 69.06]	12.05	[−10.99, 35.09]	17.17	[−7.06, 41.39]	10.95	[−13.32, 35.23]	*161*	*193*
3−4	10.8	[9.1, 12.7]	261.4	*10.91*	58.75	[28.69, 88.80]	14.25	[−11.46, 39.96]	20.23	[−6.45, 46.92]	13.98	[−12.80,40.76]	*179*	*196*
5+	11.3	[9.5, 13.3]	268.5	*11.10*	62.72	[33.56,91.87]	15.83	[−9.92, 41.58]	18.86	[−7.42,45.15]	12.60	[−14.15, 39.35]	*184*	*206*
						p <.0001		p =.232		p =.466		p =.702		
Concurrency&														
No	86.7	[84.6, 88.5]	220.3	*3.43*	-	-	-	-	-	-	-	-	*1338*	*1580*
Yes	13.3	[11.5, 15.4]	262.7	*9.58*	40.30	[21.20, 59.41]	24.24	[7.48, 41.01]	22.27	[5.46, 39.09]	20.87	[4.47, 37.26]	*226*	*243*
						p <.0001		p =.0046		p =.0095		p =.0127		
Paid for sex														
No	96.3	[95.0, 97.4]	225.2	*3.41*	-	-	-	-	-	-	-	-	*1512*	*1771*
Yes	3.7	[2.6, 5.0]	230.3	*19.59*	5.12	[−33.88, 44.12]	5.87	[−30.34,42.07]	5.31	[−31.67, 42.30]	10.66	[−24.62, 45.94]	*56*	*67*
						p =.7969		p =.7506		p =.778		p =.554		
Same-sex partner[s]														
No	97.0	[96.0, 97.8]	222.8	*3.40*	-	-	-	-	-	-	-	-	*1537*	*1823*
Yes	3.0	[2.2, 4.0]	251.9	*14.74*	25.12	[−5.01,55.24]	24.8	[0.47, 49.12]	20.88	[4.00, 45.76]	22.30	[−0.75, 45.34]	*58*	*56*
						p =.102		p =.0457		p =.0999		p =.058		
*Sexual behavior, lifetime*														
Number of sexual partners#														
0/1	14.7	[12.6, 17.1]	229.8	*7.72*	*-*	-	-	-	-	-	-	-	*237*	*265*
2	8.5	[7.0, 10.3]	224.2	*11.40*	1.36	[−25.55, 28.27]	7.23	[−14.60, 29.05]	8.01	[−13.0, 29.02]	4.73	[−15.71, 25.18]	*129*	*153*
3−4	15.7	[13.6, 18.2]	232.7	*7.49*	8.33	[−12.17, 28.84]	9.28	[−8.91, 27.46]	10.68	[−7.88, 29.23]	9.72	[−9.00, 28.44]	*237*	*285*
5−9	25.4	[22.9, 28.0]	231.5	*6.47*	6.49	[−12.14, 25.12]	15.56	[−1.00, 32.13]	17.23	[0.62, 33.85]	17.74	[1.04−34.44]	*375*	*459*
10+	35.8	[32.8, 38.8]	218.4	*5.31*	−7.33	[−24.45, 9.79]	5.12	[−10.61,20.85]	4.65	[−11.35, 20.64]	6.25	[−9.62, 22.12]	*566*	*647*
						p =.330		p =.404		p =.254		p =.252		
Ever had same sex experience with genital contact														
No	93.3	[91.7, 94.6]	223.3	*3.46*	-	-	-	-	-	-	-	-	*1472*	*1752*
Yes	6.7	[5.4, 8.3]	229.2	*10.69*	4.31	[−17.57, 26.19]	6.40	[−11.15,23.95]	2.66	[−15.12, 20.44]	0.95	[−15.72, 17.62]	*123*	*126*
						p =.699		p =.475		p =.951		p =.911		
**Sexual attraction**														
Ever felt sexually attracted to: Opposite sex only	93.2	[91.8, 94.3]	222.1	*3.45*									*1437*	*128*
Any same-sex attraction	6.8	[5.7, 8.2]	239.0	*12.15*	9.78	[−10.70, 30.26]	7.81	[−8.37, 23.99]	3.29	[−12.90, 19.45]	0.59	[−14.76, 15.94]	*155*	*1877*
						p =.349		p =. 3439		p =.690		p =.940		
**Attitudes**														
One night stands														
Other	82.1	[79.5, 84.3]	218.4	*3.44*	-	-	-	-	-	-	-	-	*1300*	*1544*
‘Not wrong at all’	17.9	[15.7, 20.5]	246.0	*8.20*	24.95	[8.98, 40.92]	18.52	[5.15, 31.89]	16.82	[3.69, 29.96]	14.42	[1.70, 27.13]	*298*	*338*
						p =.0022		p =.0067		p=.0121		p =.026		
Non-exclusivity in marriage														
Other	46.5	[43.4, 49.6]	222.7	*4.84*	-	-	-	-	-	-	-	-	*758*	*877*
‘Always wrong’	53.5	[50.4, 56.6]	224.2	*4.44*	−0.79	[−13.05, 11.47]	−13.10	[−23.76, −2.44]	−13.70	[−24.20,−3.19]	−10.01	[−20.07, 0.05]	*841*	*1008*
						p =.899		p =.0161		p =.0415		p =.0512		
‘Men have a naturally higher sex drive than women’														
Other	90.5	[88.7, 92.1]	223.2	*3.54*	-	-	-	-	-	-	-	-	*1450*	*1707*
Strongly agree	9.5	[7.9, 11.3]	226.1	*10.28*	2.67	[−19.20, 24.55]	−2.70	[−23.00,17.61]	−0.97	[−21.45, 19.51	2.09	[−17.90, 22.09]	*149*	*179*
						p =.811		p =.795		p =.926		p =.837		
‘It is natural for people to want sex less as they get older’														
Other	95.7	[94.3, 96.7]	223.9	*3.41*	-	-	-	-	-	-	-	-	*1527*	*1804*
Strongly agree	4.3	[3.3, 5.7]	214.5	*13.53*	−6.43	[−34.72, 21.86]	4.23	[−16.57, 25.02]	5.08	[−15.85, 26.01]	9.10	[−9.58, 27.77]	*72*	*82*
						p =.656		p =.690		p =.634		p =.339		

unwt = unweighted denominators, wt = weighted denominators. SE = standard error of mean. Denominator: all men excluding those taking medication for epilepsy or prostate disease, or who received treatment for a testicular or pituitary condition in the past year.

* Linear regression. Adjusted coeff 1 = adjusted for age and age-squared; adjusted coeff2 = adjusted for age, age-squared, season, and relationship status; adjusted coeff3 = adjusted for age, age-squared, season, relationship status, BMI, and self-reported general health;

# opposite- and/or same-sex

^ Only asked of those who had sex in the past year, those who did not have sex in the past year were excluded from the denominator. &Overlap between any partners in past 5 years

**Table 2:** Associations between mean Sal-T and sexual behaviors and sexual function among women.

													*Denominators*	
	% of sample [Wt]	95% CI	Mean Sal-T pmol/L	*SE*	Crude ratios*	95% CI	adjusted ratios* 1	95% CI	adjusted ratios* 2	95% C.I	adjusted ratios* 3	95% CI	*unwt*	*wt*
All women	100%		37.1	*0.86*									*2123*	*1899*
**Sexual function**														
Uncomfortably dry vagina for at least 3 months in past yr^														
No	86.0	[84.0, 87.8]	38.8	*0.99*	1.00	-	1.00	-	1.00	-	1.00	-	*1310*	*1252*
Yes	14.0	[12.2, 16.0]	32.6	*1.71*	0.87	[0.78, 0.97]	0.93	[0.84, 1.04]	0.92	[0.83, 1.03]	0.92	[0.84, 1.03]	*230*	*204*
					p =.0115		p =.189		p =.147		p =.154			
Lacked interest in having sex for at least 3 months in past yr^														
No	66.6	[63.8, 69.2]	40.0	*1.21*	1.00	-	1.00	-	1.00	-	1.00	-	*1029*	*969*
Yes	33.4	[30.8, 36.2]	33.8	*1.13*	0.88	[0.81, 0.97]	0.91	[0.83, 0.99]	0.92	[0.84, 1.00]	0.92	[0.84, 1.00]	*511*	*487*
					p =.0070		p =.0312		p =.0554		p =.0592			
Lacked enjoyment when having sex for at least 3 months in past yr^														
No	87.6	[85.5, 89.4]	38.3	*0.97*	1.00	-	1.00	-	1.00	-	1.00	-	*1339*	*1275*
Yes	12.4	[10.6, 14.5]	35.0	*1.83*	0.95	[0.85, 1.06]	0.95	[0.86. 1.06]	0.95	[0.86, 1.06]	0.95	[0.86, 1.06]	*201*	*181*
					p =.337		P =.383		p =.373		p =.390			
Distressed or worried about sex life: agree strongly/agree														
No	88.7	[86.7, 90.4]	37.1	*0.91*	1.00	-	1.00	-	1.00	-	1.00	-	*1858*	*1622*
Yes	11.3	[9.6, 13.3]	40.6	*2.95*	1.05	[0.91, 1.22]	1.02	[0.89, 1.16]	1.02	[0.89, 1.17]	1.02	[0.90, 1.17]	*210*	*207*
					p =.505		p =.810		p =.787		P =.747			
Overall sexual function^														
Normal	79.0	[76.6, 81.3]	38.4	*1.04*	1.00	-	1.00	-	1.00	-	1.00	-	*1209*	*1156*
Low	21.0	[18.7, 23.4]	36.2	*1.66*	0.92	[0.83, 1.02]	0.96	[0.87, 1.06]	0.95	[0.87, 1.05]	0.95	[0.87, 1.05]	*336*	*306*
					p =.132		p =.398		p =.334		p =.353			
**Sexual behavior**														
*Masturbation*														
Last occasion of masturbation														
In last 7 days	17.2	[15.2, 19.5]	45.3	*2.55*	1.00		1.00		1.00		1.00		*362*	*318*
Between 7 days and 4 weeks	19.2	[17.2, 21.5]	39.5	*1.91*	0.92	[0.81, 1.04]	0.93	[0.82, 1.05]	0.93	[0.83, 1.05]	0.93	[0.93, 1.05]	*396*	*355*
Between 4 weeks and 1 year	21.6	[19.5, 23.8]	34.6	*1.38*	0.79	[0.70, 0.89]	0.84	[0.74, 0.94]	0.84	[0.75, 0.95]	0.84	[0.75, 0.95]	*460*	*398*
Longer than 1 year ago/never	41.9	[39.2, 44.7]	34.2	*1.49*	0.74	[0.66, 0.83]	0.82	[0.73, 0.92]	0.83	[0.74, 0.93]	0.83	[0.74, 0.93]	*862*	*774*
					p <.0001		p =.0030		p =.0062		p =.0077			
Number of occasions of masturbation in past 7 days														
0	83.1	[81.0, 85.1]	35.5	*0.91*	0.91	[0.79, 1.04]	0.96	[0.85, 1.09]	0.97	[0.85, 1.10]	0.97	[0.86, 1.10]	*1718*	*1526*
1	8.8	[7.5, 10.2]	40.5	*3.26*	1.00	-	1.00	-	1.00	-	1.00	-	*186*	*161*
2+	8.1	[6.8, 9.7]	48.4	*3.48*	1.28	[1.07, 1.52]	1.23	[1.04, 1.46]	1.24	[1.05, 1.46]	1.24	[1.05, 1.46]	*172*	*149*
					p <.0001		p =.0005		p =.0008		p =.0009			
*Sexual behavior in the past 4 weeks*														
Number of occasions of sex#														
0−2	59.2	[56.5, 61.8]	35.7	*1.20*	1.00	-	1.00	-	1.00	-	1.00	-	*1258*	*1048*
3−4	15.8	[14.0, 17.9]	36.6	*2.13*	1.04	[0.93, 1.17]	0.97	[0.87, 1.09]	0.97	[0.86, 1.08]	0.96	[0.86−1.08]	*286*	*281*
5+	25.0	[22.5, 27.7]	39.3	*1.50*	1.18	[1.08, 1.28]	1.03	[0.94, 1.13]	1.03	[0.93, 1.13]	1.02	[0.92−1.13]	*440*	*443*
					p =.0011		p =.6410		p =.600		p =.602			
Vaginal sex														
No	41.3	[38.9, 43.7]	36.2	*1.45*	1.00	-	1.00	-	1.00	-	1.00	-	*974*	*766*
Yes	58.7	[56.3, 61.1]	37.7	*1.05*	1.07	[1.00, 1.16]	0.96	[0.89, 1.04]	0.95	[0.87, 1.03]	0.94	[0.86, 1.03]	*1120*	*1089*
					p =.0597		p =.304		p =.211		p =.200			
Received oral sex#														
No	68.2	[65.7, 70.5]	35.1	*1.07*	1.00	-	1.00	-	1.00	-	1.00	-	*1463*	*1262*
Yes	31.8	[29.5, 34.3]	41.5	*1.40*	1.20	[1.11, 1.30]	1.07	[0.99, 1.16]	1.07	[0.99, 1.17]	1.07	[0.99, 1.16]	*628*	*589*
					p <.0001		p =.0937		p =.0905		p =.0995			
Gave oral sex#														
No	66.2	[63.8, 68.6]	35.1	*1.07*	1.00	-	1.00	-	1.00	-	1.00	-	*1421*	*1226*
Yes	33.8	[31.4, 36.2]	41.0	*1.35*	1.20	[1.11, 1.30]	1.07	[0.99, 1.16]	1.07	[0.99, 1.16]	1.07	[0.99, 1.16]	*671*	*625*
					p <.0001		p =.0777		p =.0927		p =.101			
Anal sex#														
No	96.7	[95.6, 97.6]	36.8	*0.86*	1.00	-	1.00	-	1.00	-	1.00	-	*2037*	*1792*
Yes	3.3	[2.4, 4.4]	46.7	*5.00*	1.24	[0.98, 1.58]	1.18	[0.94, 1.49]	1.17	[0.92, 1.48]	1.16	[0.91−1.48]	*55*	*61*
					p =.0773		p =.163		p =.199		p =.209			
Genital contact without intercourse#														
No	58.5	[55.9, 61.0]	35.8	*1.11*	1.00	-	1.00	-	1.00	-	1.00	-	*1306*	*1088*
Yes	41.5	[39.0, 44.1]	39.5	*1.44*	1.15	[1.06, 1.24]	1.03	[0.95, 1.11]	1.02	[0.94, 1.11]	1.02	[0.93−1.11]	*789*	*772*
					p =.0007		p =.529		p =.626		p =.663			
*Sexual behavior in the past 5 years*														
Number of sexual partners#														
0	13.1	[11.6, 14.7]	36.0	*3.01*	1.00	-	1.00	-	1.00	-	1.00	-	*361*	*239*
1	61.6	[59.2, 64.0]	35.8	*0.98*	1.14	[1.02, 1.28]	0.99	[0.89, 1.11]	1.00	[0.88, 1.14]	1.00	[0.88, 1.14]	*1177*	*1129*
2	10.4	[9.0, 12.0]	39.2	*2.43*	1.28	[1.10, 1.48]	0.98	[0.84, 1.15]	0.97	[0.83, 1.14]	0.97	[0.83, 1.14]	*222*	*190*
3−4	7.7	[6.4, 9.3]	38.0	*2.24*	1.26	[1.10, 1.46]	0.92	[0.78, 1.07]	0.93	[0.79, 1.09]	0.92	[0.79, 1.08]	*157*	*142*
5+	7.2	[5.9, 8.6]	48.4	*3.44*	1.51	[1.25, 1.83]	1.05	[0.86, 1.29]	1.04	[0.84, 1.27]	1.03	[0.84, 1.27]	*161*	*131*
					p =.0001		p =.677		p =.719		p =.731			
Concurrency&														
No	92.0	[90.5, 93.2]	36.9	*0.89*	1.00	-	1.00	-	1.00	-	1.00	-	*1906*	*1685*
Yes	8.0	[6.8, 9.5]	41.1	*2.73*	1.12	[0.98, 1.29]	0.97	[0.84, 1.11]	0.96	[0.84, 1.10]	0.96	[0.84, 1.09]	*173*	*147*
					p =.0873		p =.620		p =.548		p =.535			
Same-sex partner[s]														
No	97.2	[96.4, 97.9]	36.7	*0.87*	1.00	-	1.00	-	1.00	-	1.00	-	*2055*	*1846*
Yes	2.8	[2.1, 3.6]	50.9	*5.50*	1.35	[1.11, 1.65]	1.24	[1.03, 1.49]	1.20	[0.99, 1.44]	1.20	[1.00, 1.45]	*67*	*52*
					p =.0027		p =.0257		p =.0581		p =.0540			
*Sexual behavior, lifetime*														
Number of sexual partners#														
0/1	23.6	[21.3, 26.0]	37.9	*2.46*	1.00	-	1.00	-	1.00	-	1.00	-	*439*	*429*
2	11.7	[10.1, 13.5]	40.0	*2.63*	1.00	[0.86, 1.17]	1.02	[0.88, 1.17]	1.02	[0.89, 1.17]	1.02	[0.89, 1.17]	*234*	*213*
3−4	19.3	[17.3, 21.4]	34.2	*1.62*	0.91	[0.80, 1.02]	0.89	[0.79, 1.00]	0.89	[0.79, 0.99]	0.89	[0.79, 0.99]	*378*	*351*
5−9	25.0	[22.8, 27.3]	38.0	*1.31*	1.06	[0.96, 1.18]	0.99	[0.89, 1.09]	0.98	[0.89, 1.08]	0.98	[0.89, 1.08]	*516*	*455*
10+	20.4	[18.6, 22.3]	36.8	*1.68*	0.98	[0.87, 1.10]	0.91	[0.82, 1.01]	0.91	[0.81, 1.01]	0.90	[0.81, 1.01]	*498*	*371*
					p =.0546		p =.107		p =.111		p =.106			
Ever had same sex experience with genital contact														
No	94.4	[93.3, 95.4]	36.6	*0.89*	1.00	-	1.00	-	1.00	-	1.00	-	*1983*	*1793*
Yes	5.6	[4.6, 6.7]	44.6	*3.18*	1.22	[1.06, 1.39]	1.15	[1.01, 1.32]	1.15	[1.00, 1.31]	1.15	[1.01, 1.31]	*140*	*106*
					p =.0054		p =.0325		p =.0424		p =.0378			
**Sexual attraction**														
Ever felt sexually attracted to:														
Opposite sex only	88.1	[86.5, 89.6]	36.2	*0.92*	1.00	-	1.00	-	1.00	-	1.00	-	*1826*	*1663*
Any same-sex attraction	11.9	[10.4, 13.5]	42.6	*2.33*	1.15	[1.04, 1.27]	1.04	[0.94, 1.14]	1.03	[0.93, 1.14]	1.03	[0.93, 1.14]	*285*	*224*
					p =.0063		p =.477		p =.569		p =.530			
**Attitudes**														
One night stands														
Other	90.5	[89.0, 91.8]	36.8	*0.92*	1.00		1.00	-	1.00	-	1.00	-	*1909*	*1718*
‘Not wrong at all’	9.5	[8.2, 11.0]	40.3	*2.21*	1.11	[0.99, 1.25]	1.08	[0.97−1.20]	1.04	[0.93, 1.17]	1.04	[0.93, 1.16]	*214*	*181*
					p =.0692		p =.152		p =.453		p =.485			
Non-exclusivity in marriage														
Other	38.5	[36.0, 41.1]	34.9	*1.13*	1.00		1.00	-	1.00	-	1.00	-	*856*	*731*
‘Always wrong’	61.5	[58.9, 64.0]	38.4	*1.22*	1.05	[0.97, 1.14]	1.00	[0.93, 1.08]	1.00	[0.93, 1.07]	1.00	[0.93, 1.08]	*1266*	*1167*
					p =.203		p =.970		p =.970		p =.930			
‘Men have a naturally higher sex drive than women’														
Other	86.7	[84.7, 88.5]	36.8	*0.82*	1.00		1.00	-	1.00	-	1.00	-	*1864*	*1647*
Strongly agree	13.3	[11.5, 15.3]	39.0	*3.07*	1.04	[0.93, 1.16]	1.04	[0.94, 1.15]	1.04	[0.94, 1.15]	1.04	[0.94, 1.15]	*259*	*252*
					p =.488		p =.485		p =.464		p =.451			
‘It is natural for people to want sex less as they get older’														
Other	93.1	[91.8, 94.2]	37.6	*0.91*	1.00		1.00	-	1.00	-	1.00	-	*1972*	*1768*
Strongly agree	6.9	[5.8, 8.2]	30.5	*2.15*	0.82	[0.72, 0.94]	0.89	[0.78, 1.01]	0.90	[0.79, 1.03]	0.90	[0.79, 1.03]	*151*	*131*
					p =.0051		p =.0824		p =.120		p =.128			

unwt = unweighted denominators, wt = weighted denominators. SE = standard error of mean. Denominator is: all women excluding those taking medication for epilepsy, received treatment for an ovarian or pituitary condition or polycystic ovaries in the past year, women currently receiving HRT, or who had ever received HRT and reported a hysterectomy (to approximate oophorectomy), or who were pregnant at the time of interview.

* Ratio of geometric means, obtained from exponentiated age-adjusted linear regression coefficients of log-transformed data for women. Adjusted ratio 1 = adjusted for age and age-squared; adjusted ratio 2 = adjusted for age, age-squared, season, and relationship status; adjusted ratio 3 = adjusted for age, age-squared, season, relationship status, BMI, and self-reported general health,

# opposite- and/or same-sex

^ Only asked of those who had sex in the past year, those who did not have sex in the past year were excluded from the denominator. &Overlap between any partners in past 5 years.
